# Brucella outer membrane protein 16 induces trophoblast pyroptosis via rewiring IL-6 trans-signaling and caspase-3/GSDME axis

**DOI:** 10.1371/journal.pntd.0014510

**Published:** 2026-07-20

**Authors:** Hui Ren, Guanti Lai, Wenjie Shen, Tianhang Zhu, Heng Yang, Yongshui Fu, Chengyao Li, Tingting Li, Hailong Su, Wenjing Wang

**Affiliations:** 1 Department of Transfusion Medicine, School of Laboratory Medicine and Biotechnology, Southern Medical University, Guangzhou, China; 2 Institute of Blood Transfusion and Hematology, Guangzhou Blood Center, Guangzhou Medical University, Guangzhou, China; 3 The Key Medical Laboratory of Guangzhou, Guangzhou, China; 4 Senior Department of Obstetrics and Gynecology, The Seventh Medical Center of PLA General Hospital, Beijing, China; 5 Department of Urinary Surgery, Nanfang Hospital, Southern Medical University, Guangzhou, China; 6 Department of Transfusion Medicine, Zhujiang Hospital, Southern Medical University, Guangzhou, China; 7 Guangzhou First People’s Hospital, Guangzhou, China; 8 Department of Immunology, Zunyi Medical University, Zunyi, China; 9 Key Laboratory for Cancer Prevention and treatment of Guizhou Province, Zunyi, China; University of Connecticut College of Agriculture Health and Natural Resources, UNITED STATES OF AMERICA

## Abstract

Brucellosis is a prevalent zoonotic disease that poses a serious threat to human health, particularly during pregnancy, where infection is strongly associated with spontaneous abortion. However, the mechanisms by which Brucella infection disrupts placental function remain poorly understood. Trophoblast cells are essential for placental development and represent primary targets of Brucella infection at the maternal–fetal interface. In this study, we investigated the effects of Brucella outer membrane lipoprotein 16 (L16) on trophoblast cell fate using the human JEG-3 cell line. L16 markedly suppressed trophoblast cell proliferation and induced membrane damage, as evidenced by increased lactate dehydrogenase release. Unlike lipopolysaccharide stimulation, L16 triggered a lytic, non-apoptotic form of programmed cell death characterized by plasma membrane ballooning. Mechanistically, L16 activated caspase-3 and promoted cleavage of gasdermin E (GSDME) into its pore-forming N-terminal fragment, thereby executing GSDME-dependent pyroptosis. Silencing of caspase-3 abolished GSDME cleavage and significantly attenuated pyroptotic cell death, confirming the essential role of the caspase-3/GSDME axis. Further analysis revealed that L16 reprogrammed inflammatory and survival signaling in trophoblast cells. L16 suppressed interleukin-6 (IL-6) trans-signaling by downregulating IL-6 receptor expression and up-regulating suppressor of cytokine signaling 3 (SOCS3), leading to inhibition of the JAK2/STAT3 pathway. Concurrently, L16 inhibited PI3K/AKT signaling and reduced phosphorylation of X-linked inhibitor of apoptosis protein (XIAP), thereby releasing inhibitory constraints on caspase-3 activation. Transcriptomic profiling supported coordinated repression of IL-6/JAK/STAT3 and PI3K/AKT pathways following L16 exposure. Collectively, these findings identify L16 as a key *Brucella* virulence factor that induces trophoblast pyroptosis by rewiring IL-6 trans-signaling and survival pathways, providing new mechanistic insight into *Brucella*-associated pregnancy failure.

## Author summary

Brucellosis is an infectious disease that can be transmitted from animals to humans and is an important cause of reproductive problems in livestock and, in some cases, pregnancy complications in humans. However, it is still not fully clear how Brucella infection damages the placenta and contributes to pregnancy failure. In this study, we focused on a bacterial outer membrane protein called L16 and examined how it affects trophoblast cells, which are key cells that help form and maintain the placenta.We found that L16 impaired trophoblast cell growth and caused a destructive form of cell death in which the cell membrane becomes damaged and ruptures. Our results show that this process is driven by a specific cell death pathway involving caspase-3 and gasdermin E. We also found that L16 weakens important survival and inflammatory signaling pathways that normally help trophoblast cells maintain their function. These findings suggest that L16 may contribute to placental injury during Brucella infection and provide a clearer explanation for how brucellosis may be linked to pregnancy loss.

## Introduction

Brucellosis is one of the most prevalent zoonotic diseases worldwide and is caused by Gram-negative bacteria of the genus *Brucella* [1]. Human infection often results in a strong inflammatory response and can lead to a wide spectrum of systemic manifestations involving the nervous, circulatory, and reproductive systems [2]. Among these complications, adverse pregnancy outcomes represent a particularly severe clinical consequence. Epidemiological studies have reported that the incidence of spontaneous abortion in pregnant women with brucellosis can reach as high as 43% in endemic regions such as Saudi Arabia [3,4]. Despite its clinical significance, the cellular and molecular basis of pregnancy failure associated with *Brucella* infection is still poorly understood [5].

The placenta plays a central role in maintaining maternal–fetal immune tolerance and supporting fetal development. Trophoblast cells constitute the first cellular barrier encountered by maternal blood and are among the primary targets of *Brucella* infection within placental tissue. Proper proliferation, differentiation, and invasion of trophoblast cells are essential for implantation, placental development, and maintenance of pregnancy [6]. Disruption of these tightly regulated processes can compromise placental integrity and ultimately lead to abortion [7,8]. Programmed cell death is a fundamental mechanism in placental remodeling; however, excessive or dysregulated trophoblast cell death has been implicated in recurrent spontaneous abortion and other placental pathologies [7,9,10]. Notably, trophoblast cell death has been shown to provoke maternal inflammatory responses and promote the release of lactate dehydrogenase (LDH), further amplifying placental injury [11]. These observations suggest that pathogen-induced trophoblast dysfunction may represent a critical event in *Brucella*-associated pregnancy failure.

The outer membrane of *Brucella* contains several immunologically active components, including lipopolysaccharide (LPS), outer membrane proteins (OMPs), and phospholipids. While *Brucella* LPS is a classical ligand recognized by the innate immune system, accumulating evidence indicates that *Brucella* OMPs also play important roles in host–pathogen interactions. Among them, outer membrane lipoprotein 16 (L16) is highly conserved in *Brucella* melitensis isolates infecting humans and has been shown to elicit strong immune responses in human peripheral blood mononuclear cells [12]. In addition, *Brucella* abortus infection has been reported to induce inflammatory responses through Toll-like receptor 2 (TLR2)–dependent activation of the nuclear factor kappa B (NF-κB) signaling pathway [13,14]. However, whether specific OMPs such as L16 directly contribute to trophoblast injury and pregnancy loss has not been elucidated.

Interleukin-6 (IL-6) is a pleiotropic cytokine that plays a pivotal role in host defense and inflammatory regulation during infection. IL-6 signaling can occur through a trans-signaling mechanism involving binding of IL-6 to its specific receptor (IL-6R), which is closely associated with IL-6 secretion and inflammatory amplification [15]. Activation of IL-6 trans-signaling promotes the production of pro-inflammatory mediators through downstream JAK/STAT3 and PI3K/AKT/NF-κB signaling pathways [16]. TLR2 has been identified as an important upstream regulator of the IL-6/IL-6R signaling complex and is functionally linked to STAT3 activation [17]. Suppressor of cytokine signaling 3 (SOCS3) serves as a critical negative regulator of JAK/STAT signaling by restraining cytokine-driven signal transduction [18]. Moreover, NF-κB activation has been shown to precede and modulate STAT3 signaling in inflammatory contexts, suggesting extensive crosstalk among these pathways [19]. Dysregulation of IL-6 trans-signaling and its associated survival pathways may therefore profoundly affect trophoblast cell fate during infection.

Despite these advances, the key molecular events by which *Brucella* infection induces trophoblast cell death and leads to spontaneous abortion in humans remain unclear. In particular, whether *Brucella*-derived OMPs trigger inflammatory forms of programmed cell death, such as pyroptosis, in trophoblast cells has not been explored. Recent studies have identified gasdermin family proteins, particularly gasdermin E (GSDME), as key executors of non-canonical pyroptosis downstream of caspase-3 activation [20]. In this study, we systematically investigated the effects of *Brucella* outer membrane lipoprotein 16 on trophoblast cell survival and inflammatory signaling. Our findings reveal that L16 induces caspase-3/GSDME–dependent pyroptosis in trophoblast cells by reprogramming IL-6 trans-signaling and associated JAK/STAT3, PI3K/AKT, and NF-κB pathways. These results provide new mechanistic insights into the pathogenesis of *Brucella*-induced pregnancy failure and identify potential molecular targets for therapeutic intervention.

## Materials and methods

### Cell culture

JEG-3 cells were purchased from the American Type Culture Collection. They were cultured in high-glucose Dulbecco’s modified Eagle medium (Gibco BRL, Grand Island, NY, USA) supplemented with 10% fetal bovine serum (HyClone, Thermo Scientific, Logan, UT, USA) and 1% penicillin/streptomycin (Gibco, Carlsbad, CA, USA). All cells were maintained in a humidified incubator at 37°C with 5% CO2 and routinely checked for mycoplasma contamination.

### Construction, expression and purification of recombinant lipoprotein 16 (OMP16) of *B. melitensis*

The full-length lipoprotein OMP16 (L16) (pET-28a (+) - L16) plasmid and unlipidated OMP16 (U16) (pET-28a (+) - U16) plasmid, which has the signal peptide and lipid core removed. The plasmid were transformed into the Escherichia coli BL21 (DE3) competent cells, respectively. Pick single colonies and inoculate them into 3 ml of LB liquid medium containing potassium ions (K+), and then expand the culture for induction expression. Re-suspend the whole bacteria in 200 µl of phosphate buffered saline (PBS) and lysed them using an ultrasonic disruptor. Purify the recombination proteins using a nickel column and a gradient imidazole elution method. The purified protein is dialyzes in PBS buff er for 24 h and stored at -80°C.

### MTT assay

Following L16 stimulation, the cell proliferation rate was measured using an MTT Assay Kit (MultiSciences, Hangzhou, China). JEG-3 cells were seeded in 96-well plates and treated with L16 at concentrations of 1, 2, and 5 μg/mL, respectively. After incubation with an MTT solution for 4 h at 37°C, 100 µL formazan solution was added. Absorbance was measured at 570 nm using an enzyme-labeled instrument (Epoch, Bio Tek Instruments Inc., Winooski, VT, USA).

### LDH release assay

LDH released from the treated JEG-3 cells was quantified with LDH-Glo Cytotoxicity Assay (Promega, USA). After adding 50 µL of termination solution, the level of LDH released in the 96-well plate was measured at 492nm by an enzyme-labeled instrument (Epoch, Bio Tek Instruments Inc., Winooski, VT, USA).

### Signaling pathway inhibit assay

The chemical reagents QNZ (Selleck Chemicals, Shanghai, China) was used specifically to inhibit NFκB signaling pathways. The QNZ was add into JEG-3 cells medium for 24 h before L16 incubation.

### Flow cytometry

To explore cell death in different groups, all cells were collected into 1.5 mL centrifuge tubes after co-incubation with the test proteins, followed by centrifugation at 200 g for 5 min. Then, the cell pellets were resuspended in 500 µl of 1 × binding buffer and adjusted to a final concentration of 5 × 105 cells/mL. Afterward, 100 µl of cell suspension of each group was supplemented with 5 µL of PE Annexin V and 7-amino-actinomycin (7-AAD) labeled with APC (BD Pharmingen, San Diego, USA), respectively. After incubation at room temperature for 15 min, 400 µl of binding buffer was added, and analysis was conducted using flow cytometry. The data were analyzed by FlowJo Software v10.0.

### Western blot

Prepared protein samples were separated by electrophoresis on 12% sodium dodecyl sulfate-polyacrylamide gel electrophoresis and subsequently transferred to a polyvinylidene fluoride membrane (Millipore, Billerica, USA), as previously detailed [21,22]. Then, the membrane was blocked for 2 h and washed in Tris buffered saline with 0.05% Tween-20. The membrane was incubated with specific primary antibodies at 4°C overnight. The primary antibodies used with working dilutions included: Caspase-3, Cleaved Caspase-3, Toll-like Receptor 2 (TLR2), IL-6Rα/CD126, Phosphoinositide 3-kinase (PI3K), Protein kinase B (AKT), Phosphorylated AKT (P-AKT), Suppressor of Cytokine Signaling-3 (SOCS3), Janus Kinase-2 (JAK2), Signal Transducer and Activator of Transcription-3 (STAT3), Phosphorylated STAT3 (P-STAT3), Phosphorylated X-linked inhibitor of apoptosis protein (P-XIAP), GAPDH and Tubulin were purchased from Cell Signaling Technology (Danvers, USA). Gasdermin E (GSDME) was purchased from Abcame (Cambridge, USA). XIAP was purchased from ImmunoWay Biotechnology Company (TX, USA), Following three washes, the membrane was incubated with secondary antibodies at room temperature for 1 h. The secondary antibodies included Goat Anti Rabbit IgG (H+L) (horseradish peroxidase) (ImmunoWay Biotechnology Company) and Anti-mouse IgG, Horseradish Peroxidase-linked Antibody (Cell Signaling Technology). Protein bands were visualized using enhanced chemiluminescence reagents (Millipore, Billerica, MA, USA).

### Gene silencing

Caspase-3 small interfering RNA (siRNA) was commercially synthesized, and a non-targeting RNA sequence of Gordius was used as a negative control (NC) siRNA (RiboBio, Guangzhou, China). The siRNA was transfected into THP-1 cells using X-tremeGENE siRNA Transfection Reagent (Roche, Switzerland). After 72 h transfection, the cells were incubated with L16, followed by analysis using Western blot. The oligonucleotide sequence of the caspase-3 siRNA was GACGCTACTTTTCATGCAA. The RNA sequence of Gordius was used as siNC control.

### RNA isolation and RNA-Sequence

RNA extraction with TRIzol reagent (Invitrogen, Carlsbad, USA) and quality inspection of purity and integrity by Nanodrop. The transcriptome samples of L16-induced cells and control cells were sequenced commercially in by Guangzhou Omicsmart Laboratory Center (Guangzhou, China). To investigate the differences between different subgroups, the Kyoto Encyclopedia of Genes and Genomes (KEGG) pathway enrichment analysis, Reactome Enrichment Analysis and Gene Set Enrichment Analysis (GSEA) were performed. The protein-protein interaction (PPI) network of differentially expressed genes (DEGs) related to pyroptosis was analyzed using the Search Tool for the Retrieval of Interacting Genes/Proteins database (https://string-db.org/) of known and predicted PPIs.

### Statistical analysis

Statistical analysis was performed on data obtained from at least three independent experiments, each conducted in triplicate. GraphPad Prism version 6.0 (GraphPad Software, Inc., La Jolla, CA, USA) was used for all statistical analyses.

Data are presented as mean ± standard error of the mean (SEM). Statistical analyses were performed using GraphPad Prism (version 10.1.2). Comparisons between two independent groups were conducted using Student’s t-test. For comparisons among three or more groups, one-way analysis of variance (ANOVA) followed by Bonferroni post hoc tests was applied. Two-way ANOVA was used to evaluate the effects of two independent variables and their interaction where appropriate.All experiments were performed with at least three independent biological replicates. A P-value < 0.05 was considered statistically significant.

## Results

### L16 selectively suppresses trophoblast viability and induces membrane damage independent of LPS-like cytotoxicity

**Fig 1:**
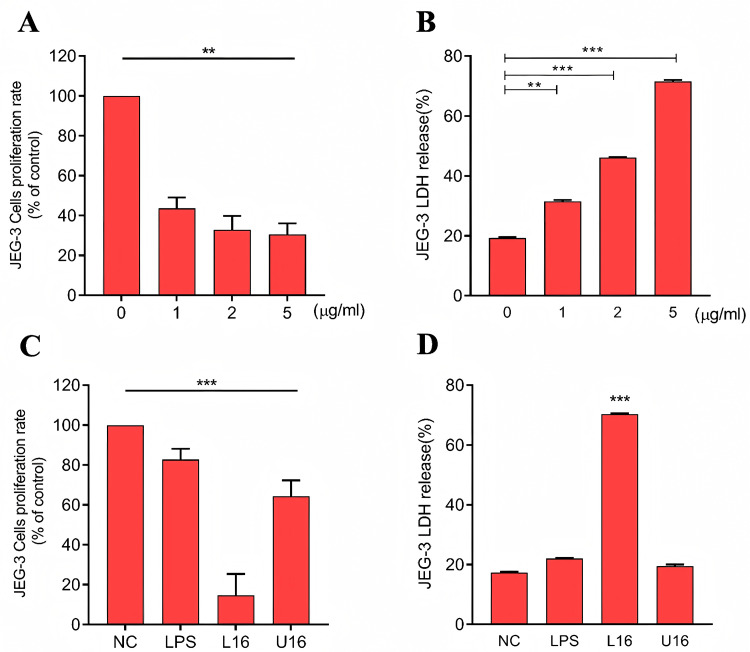
L16 selectively suppresses trophoblast cell proliferation and induces membrane damage. **(A–B)** JEG-3 cells were treated with increasing concentrations of Brucella outer membrane lipoprotein 16 (L16; 0–5 μg/mL) for 24 h, followed by assessment of cell proliferation using the MTT assay (A) and membrane damage using the lactate dehydrogenase (LDH) release assay **(B)**. **(C–D)** Comparison of cell proliferation (C) and LDH release (D) in JEG-3 cells after 24 h incubation with 5 μg/mL lipopolysaccharide (LPS), L16, or U16.Data demonstrate a dose-dependent inhibitory effect of L16 on trophoblast viability and a marked increase in membrane permeability compared with LPS and U16.

JEG-3 cells were used as an in vitro model to evaluate the effects of outer membrane protein 16 (OMP16; L16 or U16) on trophoblast viability. Cells were treated with increasing concentrations of L16 (1, 2, and 5 μg/mL) for 24 h, followed by MTT and LDH release assays. L16 reduced cell viability in a dose-dependent manner, accompanied by a marked increase in LDH release, indicating compromised membrane integrity (Fig 1A and 1B).

To determine whether this cytotoxicity resembled canonical LPS-mediated inflammatory injury, JEG-3 cells were treated with LPS (5 μg/mL), L16, or U16 for 24 h. L16 treatment resulted in a profound reduction in cell proliferation (14.7%), which was significantly lower than that observed following LPS (82.7%) or U16 (64.4%) stimulation (Fig 1C, *P* < 0.001). Consistently, LDH release was markedly elevated in the L16-treated group (70.30%) compared with the LPS (22.07%) and U16 (19.50%) groups (Fig 1D, *P* < 0.001). These findings indicate that L16 induces a distinct and selective cytotoxic response in trophoblast cells that is independent of classical LPS-like toxicity.

### L16 induces a lytic, non-apoptotic form of programmed cell death in JEG-3 cells

**Fig 2:**
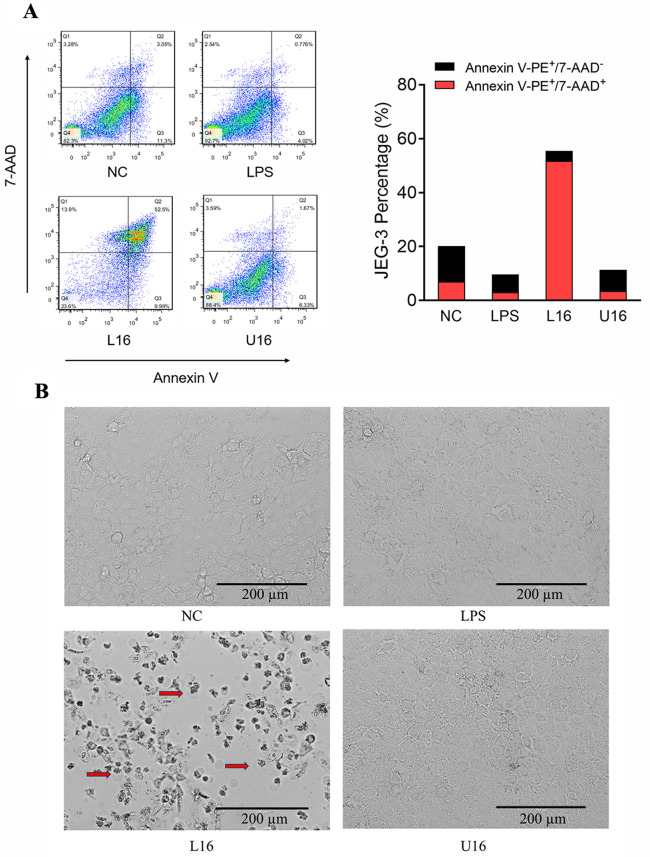
L16 induces lytic programmed cell death with pyroptotic features in JEG-3 cells. **(A)** Flow cytometric analysis of cell death in JEG-3 cells treated with LPS, L16, or U16 for 24 h using PE Annexin V/7-AAD staining. Annexin V^+^/7-AAD⁺ cells represent lytic programmed cell death. **(B)** Representative phase-contrast images showing morphological changes in JEG-3 cells following 24 h stimulation with LPS, L16, or U16. Prominent plasma membrane ballooning and cellular swelling, characteristic of pyroptosis, were observed exclusively in L16-treated cells. Scale bar, 200 μm.

To characterize the mode of cell death induced by L16, Annexin V/7-AAD staining was performed following treatment with LPS, L16, or U16 for 24 h. Notably, L16 stimulation led to a substantial increase in Annexin V ⁺ /7-AAD⁺ double-positive cells, exceeding 50% of the total cell population (Fig 2A, *P* < 0.001). In contrast, LPS- and U16-treated cells exhibited only minimal Annexin V ⁺ /7-AAD⁺ populations (0.776% and 1.67%, respectively).

Morphological examination further supported these findings. JEG-3 cells exposed to L16 displayed prominent plasma membrane ballooning and cellular swelling, hallmarks of lytic programmed cell death (Fig 2B). Such morphological alterations were not observed in cells treated with LPS or U16. Together, these results suggest that L16 induces a non-apoptotic, membrane-disruptive form of programmed cell death in trophoblast cells.

### Caspase-3 activation mediates GSDME cleavage to execute L16-induced pyroptosis

**Fig 3:**
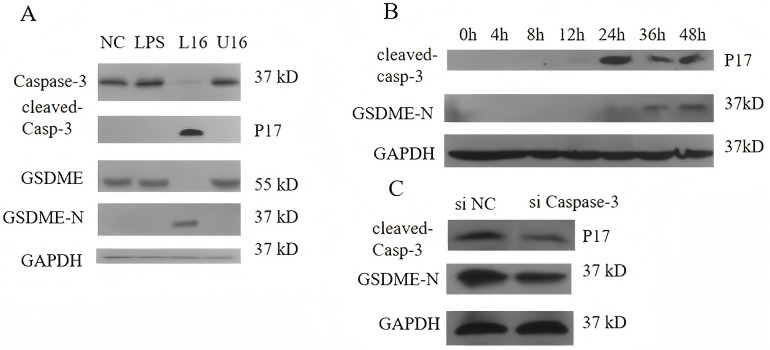
L16 induces caspase-3–dependent cleavage of GSDME to execute pyroptosis. **(A)** Western blot analysis of full-length and cleaved caspase-3, full-length gasdermin E (GSDME-FL), and cleaved N-terminal GSDME fragment (GSDME-N) in JEG-3 cells treated with LPS, L16, or U16 for 24 h. **(B)** Time-course analysis of cleaved caspase-3 and GSDME-N expression in JEG-3 cells treated with L16 for the indicated times (0–48 h). **(C)** Western blot analysis of cleaved caspase-3 and GSDME-N following caspase-3 siRNA transfection for 24h and subsequent L16 stimulation for 24h. A non-targeting siRNA derived from Gordius sequence was used as the negative control (siNC). GAPDH served as a loading control.

Gasdermin E (GSDME) has been identified as a key executor of non-canonical pyroptosis following cleavage by activated caspase-3. To determine whether this pathway is involved in L16-induced cell death, the expression of, caspase-3 and GSDME was examined in JEG-3 cells after 24 h stimulation with LPS, L16, or U16. Western blot analysis revealed that L16 robustly induced the cleavage of full-length caspase-3 (37 kDa) into its activated p17 fragment (Fig 3A). While basal expression of full-length GSDME was detected in untreated cells, L16 stimulation resulted in the disappearance of full-length GSDME and the emergence of the cleaved N-terminal fragment (GSDME-N), indicative of pyroptotic execution. In contrast, neither cleaved caspase-3 nor GSDME-N was detected in cells treated with LPS or U16 (Fig 3A).

Time-course analysis demonstrated that caspase-3 activation occurred as early as 12 h following L16 exposure, whereas GSDME-N accumulation became evident at 24 h and increased in a time-dependent manner (Fig 3B). These data indicate that L16 induces a caspase-3–dependent, GSDME-mediated pyroptotic program in trophoblast cells.

### Genetic inhibition of caspase-3 abrogates GSDME-dependent pyroptosis in trophoblast cells

To confirm the requirement of caspase-3 in L16-induced GSDME-mediated pyroptosis, JEG-3 cells were transfected with caspase-3–specific siRNA prior to L16 stimulation. Efficient knockdown of caspase-3 was confirmed by Western blot analysis (Fig 3C).

Silencing of caspase-3 markedly reduced the levels of cleaved caspase-3 (p17) and abolished the generation of GSDME-N following L16 treatment (Fig 3C). These findings provide direct genetic evidence that caspase-3 activation is indispensable for GSDME cleavage and the execution of L16-induced pyroptosis in trophoblast cells.

### L16 suppresses IL-6 trans-signaling and reprograms JAK/STAT3 activity in trophoblast cells

**Fig 4:**
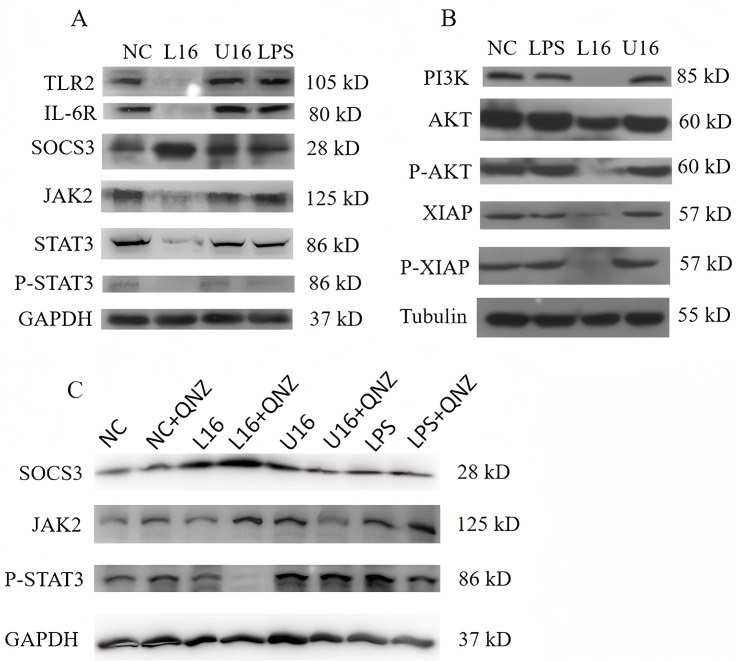
L16 reprograms IL-6 trans-signaling and PI3K/AKT survival pathways in trophoblast cells. **(A)** Western blot analysis of IL-6 receptor (IL-6R), toll-like receptor 2 (TLR2), suppressor of cytokine signaling 3 (SOCS3), JAK2, total STAT3, and phosphorylated STAT3 (P-STAT3) in JEG-3 cells treated with LPS, L16, or U16 for 24 h. **(B)** Western blot analysis of PI3K, AKT, phosphorylated AKT (P-AKT), X-linked inhibitor of apoptosis protein (XIAP), and phosphorylated XIAP (P-XIAP) under the same conditions. **(C)** JEG-3 cells were co-treated with an NF-κB inhibitor prior to L16 stimulation, followed by detection of JAK2 and STAT3 expression. GAPDH or Tubulin was used as a loading control.

To explore the upstream inflammatory signaling events associated with L16-induced pyroptosis, key components of the IL-6 trans-signaling pathway were examined. Compared with the NC, LPS, and U16 groups, L16 treatment markedly down-regulated the expression of TLR2 and IL-6 receptor (IL-6R) in JEG-3 cells (Fig 4A).

Concomitantly, L16 stimulation up-regulated the expression of suppressor of cytokine signaling 3 (SOCS3), leading to reduced expression of JAK2, total STAT3, and phosphorylated STAT3 (p-STAT3) (Fig 4A). These results indicate that L16 selectively suppresses IL-6 trans-signaling and disrupts downstream JAK/STAT3 activation in trophoblast cells.

### Inhibition of the PI3K/AKT–XIAP survival axis licenses caspase-3 activation upon L16 stimulation

Given the established role of PI3K/AKT signaling in cell survival, we next examined whether this pathway contributes to L16-induced trophoblast cell death. Western blot analysis showed that L16 treatment significantly reduced the expression of PI3K and AKT compared with NC, LPS, and U16 groups (Fig 4B).

Notably, phosphorylation of X-linked inhibitor of apoptosis protein (XIAP), a key AKT-regulated inhibitor of caspase-3, was markedly decreased following L16 stimulation. These findings suggest that L16 suppresses the PI3K/AKT–XIAP survival axis, thereby lowering the activation threshold for caspase-3 and facilitating downstream GSDME-mediated pyroptosis.

### NF-κB inhibition cooperates with IL-6/JAK signaling suppression to amplify L16-induced inflammatory reprogramming

To further clarify the interaction between NF-κB and JAK/STAT signaling in response to L16, JEG-3 cells were treated with the NF-κB inhibitor QNZ prior to L16 stimulation. Inhibition of NF-κB signaling further suppressed the expression of JAK2 and STAT3 compared with L16 treatment alone (Fig 4C).These results suggest that NF-κB signaling cooperates with IL-6/JAK/STAT3 pathways to regulate inflammatory responses in trophoblast cells, and that L16 amplifies inflammatory reprogramming through coordinated suppression of these signaling networks.

### Transcriptomic profiling reveals coordinated repression of survival and immune signaling pathways following L16 exposure

**Fig 5:**
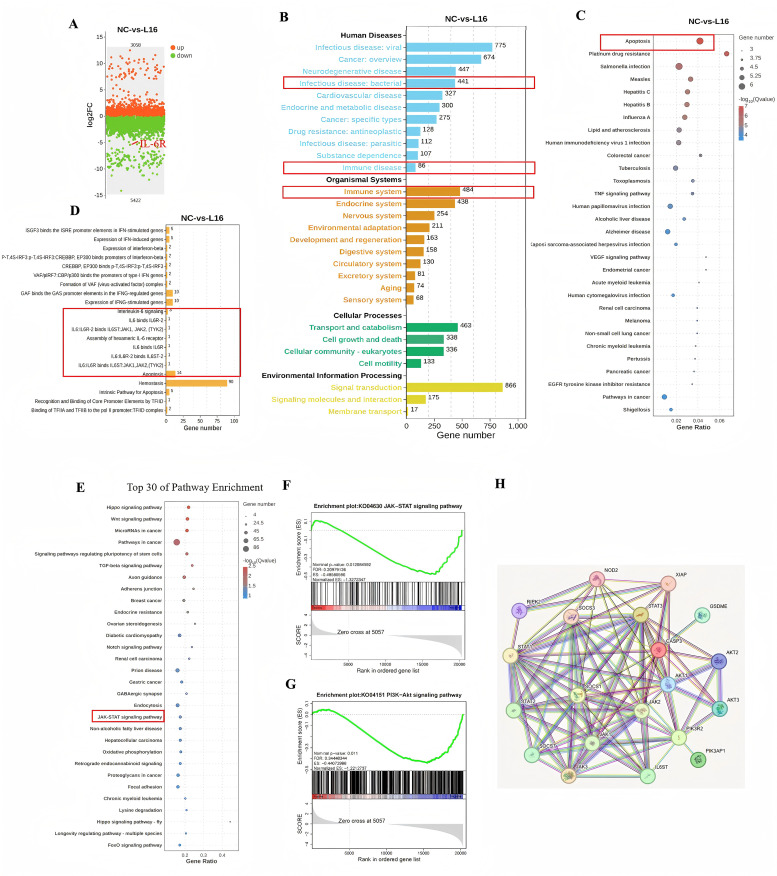
Transcriptomic profiling reveals coordinated repression of immune and survival signaling pathways following L16 exposure. **(A)** Scatter plot showing differentially expressed genes (DEGs) between L16-treated and negative control JEG-3 cells after batch effect correction (n = 3). Orange dots represent upregulated genes, and green dots represent downregulated genes. **(B)** KEGG pathway enrichment analysis of DEGs. **(C)** Bubble plot showing KEGG pathway gene counts, with selected pathways highlighted. **(D)** Reactome pathway enrichment analysis, highlighting IL-6-related signaling and apoptosis-associated pathways.**(E)** Bubble plot of KEGG pathway enrichment. **(F–G)** Representative gene set enrichment analysis (GSEA) plots demonstrating downregulation of the JAK-STAT and PI3K-AKT signaling pathways in L16-treated cells.**(H)** Protein–protein interaction (PPI) network of DEGs related to pyroptosis, JAK/STAT3, and PI3K/AKT signaling constructed using the STRING database.

To determine whether L16 induces global transcriptional reprogramming consistent with the observed signaling alterations, RNA sequencing was performed on JEG-3 cells. Differential expression analysis identified 5,422 downregulated genes and 3,058 upregulated genes following L16 treatment (fold change ≥ 1.0, *P* < 0.05), with IL-6R among the significantly downregulated transcripts (Fig 5A). KEGG pathway enrichment analysis revealed significant enrichment of genes related to infectious diseases, immune system processes, apoptosis, and the JAK–STAT signaling pathway (Fig 5B, 5C, and 5E). Reactome analysis further identified IL-6–related signaling modules and apoptosis-associated pathways as prominently affected by L16 treatment (Fig 5D). Gene set enrichment analysis confirmed coordinated down regulation of the JAK–STAT and PI3K–AKT signaling pathways in the L16 group (Fig 5F and 5G; fold change ≥ 2.0, *P* < 0.05).

### Protein–protein interaction network identifies caspase-3/GSDME as central hubs linking immune suppression to cell fate execution

To integrate transcriptomic changes with protein-level signaling events, differentially expressed immune-related genes were used to construct a protein–protein interaction (PPI) network. Twenty key genes were identified, including CASP3, GSDME, XIAP, STAT1/2/3, SOCS family members, JAK kinases, AKT isoforms, and PI3K regulatory subunits (Fig 5H).Within this network, caspase-3 and GSDME emerged as central hubs connecting suppressed immune signaling pathways to execution of programmed cell death. These results support a model in which L16-driven immune signaling repression converges on caspase-3/GSDME-mediated pyroptosis to determine trophoblast cell fate.

## Discussion

Human brucellosis is a systemic zoonotic disease that frequently involves the nervous, circulatory, and reproductive systems, with placental infection representing a major pathological hallmark during pregnancy [23]. Clinical and epidemiological studies have consistently shown that *Brucella* infection is strongly associated with adverse pregnancy outcomes, including spontaneous abortion and stillbirth, particularly during early gestation [5]. The reported incidence of pregnancy loss in women with brucellosis ranges from 31% to 46%, which is markedly higher than that observed in the general pregnant population [24,25]. However, the cellular and molecular mechanisms by which *Brucella* infection disrupts placental homeostasis remain incompletely understood.

Trophoblast cells play a central role in placental development, immune tolerance, and maternal–fetal exchange. Physiological trophoblast apoptosis is tightly regulated during normal placentation, whereas excessive or dysregulated cell death has been implicated in recurrent spontaneous abortion and other placental pathologies [26,27]. Elevated apoptotic markers in trophoblasts have been reported in pathological placentas, underscoring the importance of maintaining a balance between trophoblast proliferation and survival [28,29]. In the present study, we demonstrate that *Brucella* outer membrane protein 16 (L16) profoundly disrupts trophoblast cell homeostasis by inducing pyroptotic cell death rather than classical apoptosis, thereby providing a potential mechanistic link between *Brucella* infection and pregnancy failure.

Pyroptosis is a lytic and highly inflammatory form of programmed cell death, distinct from apoptosis, and is increasingly recognized as a critical determinant of tissue damage during infection. Recent studies have revealed that gasdermin E (GSDME) can be cleaved by activated caspase-3 at amino acid residues 267–270, thereby converting apoptotic signaling into pyroptotic execution [30,31]. In this study, L16 stimulation robustly activated caspase-3 and promoted the cleavage of full-length GSDME into its pore-forming N-terminal fragment (GSDME-N) in JEG-3 trophoblast cells (Fig 3A). Importantly, genetic silencing of caspase-3 markedly attenuated GSDME cleavage and pyroptotic cell death, providing direct evidence that L16 induces a caspase-3–dependent, GSDME-mediated pyroptosis pathway (Fig 3C). These findings identify a non-canonical pyroptotic mechanism operating in trophoblast cells and expand the functional relevance of the caspase-3/GSDME axis beyond tumor and chemotherapy contexts.

Inflammatory signaling plays a pivotal role in regulating trophoblast fate during pregnancy. IL-6 trans-signaling, which requires activation of both JAK/STAT3 and PI3K/AKT pathways, is essential for trophoblast proliferation, survival, and immune modulation at the maternal–fetal interface [16]. SOCS3 acts as a key negative regulator of IL-6 signaling by directly inhibiting JAK kinase activity through its kinase inhibitory region (KIR), with particularly high affinity for JAK2 [18,32,33]. Dysregulation of JAK/STAT signaling has been associated with impaired immune responses and adverse pregnancy outcomes [32]. In the present study, L16 markedly downregulated IL-6 receptor expression while simultaneously upregulating SOCS3, leading to suppression of the JAK2/STAT3 signaling axis in trophoblast cells (Fig 4A). These results suggest that L16 interferes with IL-6 trans-signaling, thereby shifting trophoblast cells away from a pro-survival and immunoregulatory state.

The PI3K/AKT pathway constitutes another critical survival signaling axis in trophoblast biology. AKT activation promotes trophoblast proliferation and resistance to cell death, in part through downstream activation of NF-κB and stabilization of anti-apoptotic proteins [34,35]. Previous studies have shown that inhibition of PI3K/AKT signaling in trophoblasts contributes to recurrent spontaneous abortion [36–38]. X-linked inhibitor of apoptosis protein (XIAP), a direct downstream target of AKT, suppresses caspase-3 activation by preventing its auto-cleavage and degradation [39]. In this study, L16 selectively inhibited PI3K/AKT signaling and reduced phosphorylation of both AKT and XIAP, thereby releasing the inhibitory constraint on caspase-3 activation (Fig 4B). These findings provide a mechanistic explanation for how L16 licenses caspase-3 activation and subsequent GSDME-mediated pyroptosis in trophoblast cells.

NF-κB signaling acts as a permissive or cooperative regulator that fine-tunes IL-6/JAK/STAT3 and PI3K/AKT signaling under L16 stimulation. Pharmacological inhibition of NF-κB further suppressed JAK2 and STAT3 expression in L16-treated cells, indicating that NF-κB acts as a cooperative modulator rather than an independent driver of L16-induced signaling reprogramming (Fig 4C). This coordinated suppression of IL-6 trans-signaling, PI3K/AKT survival signaling, and NF-κB activity ultimately converges on caspase-3/GSDME-dependent pyroptotic execution.

**Fig 6:**
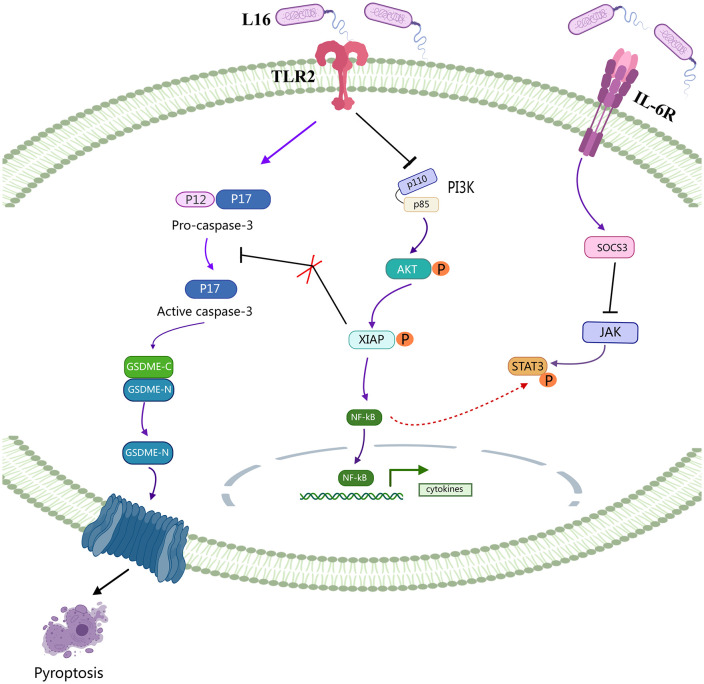
Schematic model illustrating L16-induced trophoblast pyroptosis via IL-6 trans-signaling reprogramming. Proposed mechanism by which Brucella outer membrane lipoprotein 16 (L16) induces inflammatory signaling reprogramming and pyroptosis in trophoblast cells. L16 modulates toll-like receptor 2 (TLR2) and interleukin-6 receptor (IL-6R) signaling, leading to upregulation of SOCS3 and subsequent suppression of the JAK2/STAT3 pathway. Concurrently, inhibition of IL-6 trans-signaling attenuates PI3K/AKT activity, resulting in reduced phosphorylation of XIAP. Loss of XIAP-mediated inhibition permits caspase-3 activation, which cleaves full-length GSDME into its pore-forming N-terminal fragment (GSDME-N). GSDME-N induces plasma membrane permeabilization, cell swelling, and rupture, culminating in pyroptotic cell death and release of inflammatory mediators.

In summary, this study identifies L16 as a key *Brucella* virulence factor capable of reprogramming trophoblast inflammatory and survival signaling networks. By suppressing IL-6 trans-signaling and PI3K/AKT–XIAP survival pathways, L16 lowers the threshold for caspase-3 activation and triggers GSDME-mediated pyroptosis in trophoblast cells (Fig 6). These findings advance current understanding of *Brucella* pathogenesis by revealing a previously unrecognized mechanism underlying *Brucella*-induced placental damage and spontaneous abortion in humans. Importantly, this work identifies trophoblast pyroptosis as a critical, underappreciated driver of *Brucella*-associated placental injury, providing a novel mechanistic framework for explaining pregnancy failure during *Brucella* infection. These insights expand knowledge of how *Brucella* virulence factors modulate host cell fate and inflammatory signaling, and contribute to a deep understanding of the cellular and molecular basis of *Brucella*-induced reproductive failure. Nonetheless, this study has several limitations. All mechanistic investigations were performed using in vitro cellular models, which may not fully recapitulate the complex placental microenvironment, maternal–fetal immune crosstalk, or systemic host responses that occur during natural infection. Although purified L16 was used to dissect its direct effects, the spatial organization, relative abundance, and in vivo distribution of L16 within intact Brucella may influence its biological activity during infection. Therefore, future studies using live or heat-killed bacteria, together with appropriate in vivo models, are needed to further validate the functional contribution of L16 to placental injury and vertical transmission under physiological infection conditions.
